#  Where do we go in terms of safety and quality of obstetric care in Colombia?

**Published:** 2016-03-30

**Authors:** Edgar Iván Ortiz, Jack Ludmir

**Affiliations:** 1 Profesor Titular. Departamento Obstetricia y Ginecología, Universidad del Valle, Cali, Colombia.; 2 Federación Colombiana de Obstetricia y Ginecología (Fecolsog). Bogota, Colombia.; 3 Federación Latinoamericana de Asociaciones y Sociedades de Obstetricia y Ginecología (FLASOG). Panamá, Panamá; 4 Professor and Head, Department Obstetrics and Gynecology. Pennsylvania Hospital. Perelman School of Medicine, University of Pennsylvania, Philadelphia, Pennsylvania

 Despite the great achievements in indicators of access to prenatal care and delivery care with qualified staff in Latin America, the fifth goal agreed at the Millennium Development Goals (MDG 5) of reducing maternal mortality in 75% by 2015 did not come true 1-2. In part, this can be explained by gains in coverage that do not result in safe and high quality obstetric care. 

 Extreme Maternal Morbidity (EMM), defined as a serious complication that occurs during pregnancy, childbirth and postpartum, and which threatens the life of a woman, is an anticipatory event of death. Its monitoring enables identification of actions that prevent maternal death, and therefore it is recognized as a quality tracer; Colombia, with the establishment of monitoring extreme maternal morbidity (EMM) at national level, have the opportunity to become a model country in how to improve obstetric care 3-4.

 This implies improving the skills of human resources in the analysis of cases, properly interpreting the indicators generated from their surveillance, and developing and implementing improvement plans in line with the needs of the institutions involved in obstetric care, making emphasis on quality and safety of care 5.

 The aggregate analysis of EMM cases held in the period 2007-2012 in Colombia revealed the presence of delays in obstetric care either by the lack of recognition of alarm symptoms by the patient, or the lack of adequate medical care. This information allows a country to focus on areas where care could be improved, including education of pregnant women, their families and communities; and implementation of protocols for safe obstetric care, prioritizing the most common causes of EMM, such as hypertensive disorders of pregnancy, postpartum bleeding and sepsis 6.

 What is the value of reporting EMM cases to the national health responsible if the reporting institution is not aware of their own indicators and does not use the information to establish specific programs to improve health care? 

 EMM surveillance cannot be limited only to its characterization at the level of a country, because the true potential of the event would be underestimated. It is required an ongoing analysis of cases, to develop care programs to improve quality and safety in obstetrical care, and to build indicators to monitor and evaluate the impact of improvement plans at institutional level.

 The EMM analysis and the implementation of improvement programs in care entails a reduction of maternal mortality, as it was demonstrated with compelling evidence in the significant reduction of maternal deaths in the Hospital Universitario del Valle, in Cali, Colombia, from 2005 to 2010, by monitoring and analysis of EMM, and the implementation of improvement plans 7. 

 Safe and high quality obstetric care requires commitment and leadership from both government authorities and the institutions providing obstetric care, in order to achieve a reduction of maternal deaths, a goal that is consistent with the new objectives of sustainable development 8. 

 For a country, it is mandatory to understand that EMM surveillance is not a bureaucratic requirement, which requires the implementation of real and feasible programs intended to improve the quality and safety of obstetric care.

 We think that it is necessary a new model of obstetric care, based on the centrality of the pregnant woman and her family, and not in the traditional (pyramidal) model. Women care must be comprehensive, multidisciplinary and humanized. In this new (circular) model, the woman and her family are the backbone. This model is based on four pillars: safety and quality of care, user satisfaction, commitment of all hospital staff and health personnel, and economic sustainability of the institution, which allows a constant reinvestment in care improvement 9.

 Our hope is that this new focus on the expectant mother and her family will allow us, as a country and continent, to achieve the new goals of sustainable development, in order to ensure physical and emotional well-being of women and children that ensure they reach their full potential. 
